# Effect of group versus individual antenatal care on uptake of intermittent prophylactic treatment of malaria in pregnancy and related malaria outcomes in Nigeria and Kenya: analysis of data from a pragmatic cluster randomized trial

**DOI:** 10.1186/s12936-020-3099-x

**Published:** 2020-01-29

**Authors:** Lisa Noguchi, Lindsay Grenier, Mark Kabue, Emmanuel Ugwa, Jaiyeola Oyetunji, Stephanie Suhowatsky, Brenda Onguti, Bright Orji, Lillian Whiting-Collins, Oniyire Adetiloye

**Affiliations:** 1 Jhpiego, 1615 Thames Street, Baltimore, MD 21231 USA; 2 Jhpiego Nigeria, Plot 971 Reuben Okoya Crescent, Utako, Abuja, Nigeria; 3 Jhpiego Kenya, Ring Road, 14 Riverside, Nairobi, Kenya; 4 Johns Hopkins Bloomberg School of Public Health, 615 North Wolfe Street, Baltimore, MD 21205 USA

**Keywords:** Antenatal care, Group antenatal care, Malaria, Sulfadoxine–pyrimethamine, Insecticide-treated net, Pregnancy, Prenatal care

## Abstract

**Background:**

Every year, malaria in pregnancy contributes to approximately 20% of stillbirths in sub-Saharan Africa and 10,000 maternal deaths globally. Most eligible pregnant women do not receive the minimum three recommended doses of intermittent preventive treatment with Sulfadoxine–pyrimethamine (IPTp-SP). The objective of this analysis was to determine whether women randomized to group antenatal care (G-ANC) versus standard antenatal care (ANC) differed in IPTp uptake and insecticide-treated nets (ITN) use.

**Methods:**

Prospective data were analysed from the G-ANC study, a pragmatic, cluster randomized, controlled trial that investigated the impact of G-ANC on various maternal newborn health-related outcomes. Data on IPTp were collected via record abstraction and difference between study arms in mean number of doses was calculated by *t* test for each country. Data on ITN use were collected via postpartum interview, and difference between arms calculated using two-sample test for proportions.

**Results:**

Data from 1075 women and 419 women from Nigeria and Kenya, respectively, were analysed: 535 (49.8%) received G-ANC and 540 (50.2%) received individual ANC in Nigeria; 211 (50.4%) received G-ANC and 208 (49.6%) received individual ANC in Kenya. Mean number of IPTp doses received was higher for intervention versus control arm in Nigeria (3.45 versus 2.14, p < 0.001) and Kenya (3.81 versus 2.72, p < 0.001). Reported use of ITN the previous night was similarly high in both arms for mothers in Nigeria and Kenya (over 92%). Reported ITN use for infants was higher in the intervention versus control arm in Nigeria (82.7% versus 75.8%, p = 0.020).

**Conclusions:**

G-ANC may support better IPTp-SP uptake, possibly related to better ANC retention. However, further research is needed to understand impact on ITN use.

*Trial registration* Pan African Clinical Trials Registry, May 2, 2017 (PACTR201706002254227).

## Background

Every year, 125 million pregnant women are exposed to malaria [1]. Annually, malaria in pregnancy (MIP) contributes to approximately 20% of stillbirths in sub-Saharan Africa and 10,000 maternal deaths globally [2]. In all areas with moderate to high malaria transmission in Africa, the World Health Organization (WHO) recommends integration of malaria prevention and treatment within routine antenatal care including: distribution of and counseling on routine, daily use of insecticide-treated nets (ITN); intermittent preventive treatment of MIP with Sulfadoxine–pyrimethamine (IPTp-SP) starting as early as possible in the second trimester and repeated monthly for a minimum of three doses; and prompt diagnosis and effective treatment of malaria infection [3]. To date, 39 African countries have adopted the WHO recommendations [4]. Three or more doses of IPTp has been shown to improve a range of maternal and newborn health outcomes [5]. Despite these benefits, IPTp coverage in malaria-endemic Africa remains low. As of 2017, coverage of IPTp1, IPTp2, and IPTp3 were 54%, 42%, and 22%, respectively [4]. A recent meta-analysis of national survey data found that ITN were used during pregnancy for 10.5 million of 26.9 million births across 37 countries (38.8%, 34.6–43.0) [6].

### Nigeria

Nigeria carries a particularly high burden of the world’s malaria cases (more than 50%), and has seen increasing case incidence in recent years [4], with malaria still remaining an important contributor to maternal death (estimated 11% of total) [7]. The Nigerian National Malaria Strategic Plan (NMSP) 2014–2020 is consistent with current WHO policy, i.e., IPTp at every ANC visit starting in second trimester; provision of ITN at first ANC contact; and appropriate case management of malaria illness in pregnancy [8]. The NMSP 2014–2020 targets for MIP aim for at least 80% of pregnant women to sleep inside ITNs and all eligible pregnant women attending ANC receive at least three doses of IPTp-SP via directly observed therapy (DOT) [8]. While the proportion of pregnant women who received at least two doses of SP during ANC has increased from 13% in 2010 to 37% in 2015, substantial new gains in coverage are likely limited by poor coverage of ANC contacts [9], drug stock-outs, lack of provider knowledge of IPTp protocols, and negative ANC client attitudes toward taking IPTp [10]. The 2015 Nigeria Malaria Indicator Survey found substantial prevalence of malaria in North Central Nigeria, including Nasarawa state (estimated at 36% among children age 6–59 months) [11].

### Kenya

Malaria remains a significant public health challenge in Kenya, as well, with over 70% of the population living in malaria risk areas [12]. In the Lake Endemic area of Kenya, which includes Kisumu County, prevalence of malaria by rapid diagnostic test among children age 6–59% was estimated at 33.5%. Kenya has also adopted the WHO strategy for addressing MiP: IPTp, ITN distribution and use, and MIP case management. National guidelines in Kenya state that IPTp-SP should be administered every ANC visit starting as early as possible in the 2nd trimester in the 14 counties with high malaria endemicity [13]. However, uptake of IPTp lags substantially behind ANC coverage [12]. In Kenya, approximately 56% of pregnant women receive at least two IPTp-SP doses (38% of pregnant women received at least three doses of IPTp-SP) and 58% of pregnant women slept under an ITN the previous night [12]. However, an analysis by O’Meara and colleagues found that despite access to free ITN via ANC services, households with a pregnant woman who recently attended ANC were no more likely to own an ITN than households without a pregnant woman [14].

### Antenatal care

The 2016 WHO Recommendations on Antenatal Care for a Positive Pregnancy Experience include group antenatal care (G-ANC) provided by qualified health-care professionals as an alternative to individual antenatal care for pregnant women in the context of rigorous research, depending on a woman’s preferences and provided that the infrastructure and resources for delivery of group antenatal care are available [3]. The 2015 Cochrane review on group versus conventional ANC identified no adverse outcomes associated with G-ANC and noted that women who participated in G-ANC reported higher satisfaction with care [15]. The original model of G-ANC as an intervention was designed for high-income country settings [16]. Adaptations of G-ANC for low- and middle-income countries (LMICs) have focused on increasing provision of key interventions, primarily through increasing ANC contacts, improving client-provider relationships, improving health literacy for better adherence, and increasing duration of each ANC contact. Compared to traditional ANC, G-ANC has more focus on facilitation of group interaction, knowledge sharing among peers, and addressing obstacles to care-seeking behaviours. Thus, G-ANC may represent a novel strategy to improve uptake of key MIP interventions.

## Methods

### Study design and objectives

Prospective data were analysed from the G-ANC study, a pragmatic, cluster randomized controlled trial (PACTR201706002254227) designed to evaluate the effectiveness, acceptability (among ANC clients and health care workers), and feasibility of a group antenatal care (G-ANC) model compared to routine individual ANC (i.e., local standard of care) in Nigeria and Kenya [17]. The analysis reported here aimed to determine whether study arms in each country differed with regard to IPTp-SP uptake, as well as reported use of ITN for mothers and infants.

### Participants

In the G-ANC trial, 20 clusters each in Nigeria and Kenya were randomized at a 1:1 ratio to either the intervention arm, implementing the G-ANC model, or the control arm providing standard individual ANC per country guidelines [17]. Women were enrolled from Nasarawa State, Nigeria, and Kisumu and Machakos Counties, Kenya from October 2016 to June 2017. Endline data collection was completed between May 2017 and January 2018. Participants were required to be ≤ 24 weeks gestation at time of enrollment in control sites; 20‒24 weeks gestation at time of their assigned group’s first meeting in intervention sites; ≥ 15 years old; able to provide a phone number; and to have no plans to leave the area for more than four consecutive weeks during pregnancy, or 3 months in the first year postpartum.

### Study intervention

At intervention sites, women were offered enrollment in an alternative service delivery model designed for women with low or no literacy, where those of similar gestational ages are placed into group cohorts to receive care, including clinical assessment, participatory facilitated learning, and peer support. Individual private time with an ANC provider was also included for all participants during group visits in the G-ANC model. All sites in both study arms were provided equal numbers of SP doses and ITNs prior to study initiation. Participants received IPTp-SP as DOT at study sites. In both settings, ITNs are routinely distributed to pregnant women at the first ANC visit, and administration of IPTp is part of routine ANC for pregnant women. Additional details regarding study design have been reported elsewhere [18].

In previously reported results, women assigned to the intervention arms in the G-ANC study were significantly more likely to attend four or more total ANC visits versus those in the control arms, with a larger adjusted effect size observed in Nigeria (aOR 13.30, 95% CI 7.69–22.99) compared to Kenya (aOR 7.12, 95% CI 3.91–12.97) [18]. A median increase of two ANC visits in Kenya and three in Nigeria was observed [18]. In the present analysis, data were restricted to participants in Nasarawa State, Nigeria, and Kisumu County, Kenya, where malaria is endemic.

### Data collection

Data on use of IPTp-SP were extracted from patient-held case notes (Kenya), facility-based ANC records (Nigeria), facility ANC registers, and study-specific registers. Participants completed an interviewer-administered home-based survey for recently delivered women (RDW) at 3‒6 weeks postpartum that included questions on use of ITN the previous night by mother and infant. Study staff entered data directly into REDCap™ (Research Electronic Data Capture) using tablets provided by the study. Data were analysed based on the groups to which they were randomized to compare IPTp-SP uptake and ITN use between the two study arms using t-tests and two-sample test for proportions, respectively.

Power for the trial’s primary outcome was individually maximized in each country to detect a 15-percentage point difference in the facility-based delivery rate between treatment arms. After considering cluster eligibility and sample size constraints this resulted in a power of 85% in Kenya and 80% in Nigeria. A coefficient of variation between clusters, intra-class correlation of 0.03, was used to calculate base sample sizes and then adjusted to account for 20% attrition. Final sample sizes for the full cohort were 1076 and 1026 participants for Nigeria and Kenya respectively. Separate power calculations were not carried out prospectively for comparison of malaria-related interventions or exploratory analyses of symptomatic malaria incidence in this study. While findings are reported from both Nigeria and Kenya, formal statistical comparisons between countries were not planned or undertaken.

## Results

### Participants

**Fig. 1:**
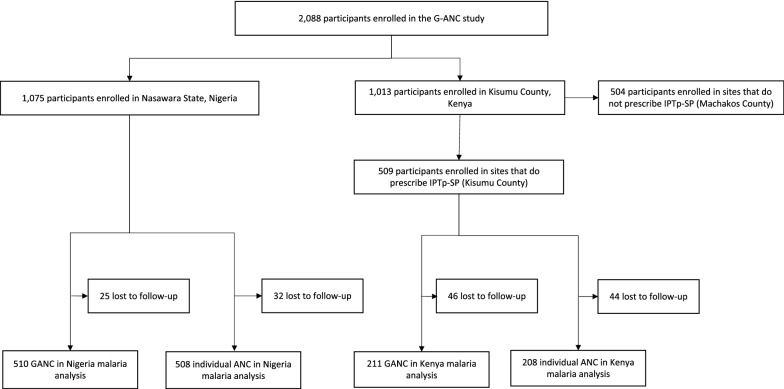
Cohort profile for malaria-related analyses

Of 2088 participants in the G-ANC trial, 1075 were enrolled in Nigeria and 1013 in Kenya respectively; in Kenya, 509 and 504 participants enrolled in Kisumu and Machakos County, respectively (Fig. 1). Data from the 1437 participants eligible for IPTp-SP use were included in the present analysis. Of the 651 excluded participants, 504 were enrolled in Machakos County, Kenya, where IPTp-SP is not recommended for ANC clients per local guidance, and 147 were lost to follow-up. Demographic characteristics of women in the two study arms for both countries are shown in Table 1. Overall, participants were similar between study arms in both countries. In Kenya, the proportion of Catholic participants was slightly higher in the intervention versus control arm (21.8% versus 17.1%, p = 0.041), as well as the proportion who used their own transportation to visit the ANC facility (62.6% vs. 52.0%, p = 0.013).

**Table 1:** Demographics and baseline characteristics of women by study arm in Nigeria and Kenya

Total	Nasarawa State, Nigeria			Kisumu County, Kenya		
	Intervention (n = 535)	Control (n = 540)	*p*-value	Intervention (n = 257)	Control (n = 252)	*p*-value
Age (years)						
15–19	47 (8.8%)	58 (10.7%)	0.663	57 (22.2%)	60 (23.8%)	0.154
20–34	453 (84.7%)	442 (81.9%)		185 (72.0%)	186 (73.8%)	
35+	35 (6.5%)	40 (7.4%)		15 (5.8%)	6 (2.4%)	
Religion (Nigeria)						
Islam	256 (47.9%)	398 (73.7%)	0.137	N/A	N/A	NA
Christian, including Catholic, other	279 (52.1%)	142 (26.3%)		N/A	N/A	
Religion (Kenya)						
Catholic	N/A	N/A	NA	56 (21.8%)	43 (17.1%)	0.041
Other christian	N/A	N/A		173 (67.3%)	192 (76.2%)	
Other	N/A	N/A		28 (10.9%)	17 (6.7%)	
Education						
None/primary/Qur’anic	280 (52.3%)	329 (60.9%)	0.494	110 (42.8%)	134 (53.2%)	0.131
Secondary/post-secondary	255 (47.7%)	211 (39.1%)		147 (57.2%)	118 (46.8%)	
Marriage						
Never married/single/widowed	9 (1.7%)	2 (0.4%)	0.054	44 (17.1%)	49 (19.4%)	0.256
Married/cohabiting	526 (98.3%)	538 (99.6%)		213 (82.9%)	203 (80.6%)	
Literacy						
Cannot read and write	232 (43.4%)	240 (44.4%)	0.919	5 (2.0%)	5 (2.0%)	0.750
Can read and write	303 (56.6%)	300 (55.6%)		252 (98.0%)	247 (98.0%)	
Parity						
Zero, never given birth before	166 (31.0%)	152 (28.1%)	0.694	87 (33.9%)	75 (29.8%)	0.841
> 0, no history complications	297 (55.5%)	306 (56.7%)		131 (50.9%)	136 (54.0%)	
> 0, history complications	72 (13.5%)	82 (15.2%)		39 (15.2%)	37 (14.7%)	
Missing data	N/A	N/A		N/A	4 (1.5%)	
Facility classification						
Rural	213 (39.8%)	162 (30.0%)	0.667	N/A	N/A	NA
Urban/Peri-urban	322 (60.2%)	378 (70.0%)		257 (100.0%)	252 (100.0%)	
Primary mode of transport						
Walk	206 (38.5%)	238 (44.1%)	0.513	94 (36.6%)	112 (44.4%)	0.013
Personal	20 (3.7%)	33 (6.1%)		161 (62.6%)	131 (52.0%)	
Public	309 (57.8%)	269 (49.8%)		2 (0.8%)	9 (3.6%)	

### Number of IPTp-SP doses received

In Nigeria, data from 1018 participants were analysed; 57 (5.3%) participants were lost to follow up. The mean number of SP doses received per woman was higher in the intervention arm compared to control (3.45 [SD 1.53] versus 2.14 [SD 1.55], p < 0.001) and women in the intervention arm were more likely to receive at least two, four, or five doses of SP, compared to control (Table 2). In Kenya, data from 419 participants from Kisumu County were analysed; 90 (17.7%) participants were lost to follow up. Study arms differed with regard to mean number of SP doses (3.81 [2.35] versus 2.72 [1.65], p = 0.001) and the proportion of women who received at least five doses of SP (46% versus 13%, p < 0.001) (Table 2).

**Table 2:** Uptake of IPTp-SP by study arm

	Intervention (n = 510)	Control (n = 508)	Difference	*p*-value
Nasarawa State, Nigeria				
Mean doses of IPTp-SP received (SD)	3.45 (1.53)	2.14 (1.55)	− 1.31	< 0.001
Doses of IPTp-SP received during ANC	n (%)	n (%)		
2+	467 (91.6)	308 (60.6%)	31.0	< 0.001
3+	347 (68.0)	183 (36.0)	32.0	0.052
4+	268 (52.6)	83 (16.3)	36.3	0.004
5+	146 (29.6)	43 (8.5)	21.1	0.034

	Intervention (n = 211)	Control (n = 208)	Difference	*p*-value
Kisumu County, Kenya				
Mean doses of IPTp-SP received (SD)	3.81 (2.35)	2.72 (1.65)	1.09	< 0.001
Doses of IPTp-SP received during ANC	n (%)	n (%)		
2+	163 (77.3)	150 (72.1)	5.2	0.590
3+	156 (73.9)	116 (55.8)	18.1	0.100
4+	131 (62.1)	77 (37.0)	25.1	0.056
5+	97 (46.0)	27 (13.0)	33.0	< 0.001

### Receipt and use of ITN by mothers and infants

In both countries, a high proportion of mothers in both study arms reported receiving ITNs during ANC (Table 3). In Nigeria, mothers in both study arms reported having received an ITN in the index pregnancy and having slept under it during the night before the survey in nearly equal proportions; in comparison, the proportion of ITN use for the infant was higher in the intervention arm (82.7% versus 75.8%, p = 0.02) (Table 3). In Kenya, reported use of ITN for the previous night before the survey was similar between study arms for both mothers and infants.

**Table 3:** Receipt and use of ITN by study arm

	Intervention (n = 510)	Control (n = 508)	Difference	*p*-value
Nasarawa State, Nigeria				
Self-reported ITN receipt and use	n (%)	n (%)		
Mother received ITN during pregnancy	455 (89.2)	505 (99.4)	− 10.2	0.100
Mother slept under ITN previous night	372 (72.9)	345 (67.9)	5.0	0.197
Infant slept under ITN previous night	422 (82.7)	385 (75.8)	6.9	0.023

	Intervention (n = 211)	Control (n = 208)	Difference	*p*-value
Kisumu County, Kenya				
Self-reported ITN receipt and use	n (%)	n (%)		
Mother received ITN during pregnancy	203 (96.7)	188 (92.2)	4.5	0.045
Mother slept under ITN previous night	198 (99.5)	191 (99.5)	0	0.978
Infant slept under ITN previous night	199 (94.3)	197 (94.7)	− 0.4	0.858

## Discussion

In this pragmatic cluster randomized controlled trial of group versus individual antenatal care, the intervention was noted to have an impact in both study countries; however, the effect appeared to be different in Nigeria versus Kenya. Among participants in Nigeria, those in the intervention arm had a higher mean number of doses of IPTp-SP received, compared to those in the control arm; in general, more women received multiple doses of IPTp-SP in the intervention versus control arm, across a range of numbers of doses. Reported use at postpartum of ITN in the previous night for infants, but not mothers, was also higher in the intervention compared to control arm in Nigeria. In Kenya, no impact was noted on maternal or infant ITN distribution or reported use. Current WHO guidelines recommend G-ANC in the context of rigorous research; these findings suggest the G-ANC service delivery model may have some potential to increase uptake of important MIP interventions, particularly IPTp coverage.

The observed increased uptake of IPTp is presumably related to the increased opportunity for administration, i.e., increased number of ANC contacts. However, the observed increase in coverage associated with the G-ANC intervention may also be related to reorganization of clinical care processes or stronger peer support for IPTp acceptability, although this was not measured formally in the trial. The lack of impact of the intervention on receipt of ITN is likely related to the timing of distribution (at the first ANC contact), which was the same in content and format for intervention and control arms.

This analysis had several strengths, including good participant retention, and DOT for IPTp. Because analysis of IPTp and self-reported ITN use was planned for this trial, robust data collection and site monitoring strategies were implemented prospectively for these variables. The use of real world health facilities as study sites suggests feasibility of this intervention outside the context of research, a theory supported by the fact that all twenty study sites have opted to sustain the G-ANC service delivery model for 2 years following withdrawal of financial support for the study.

Several important limitations should be noted. Client self-report (e.g., for ITN use) and service delivery documentation data are both subject to reporting bias, which may have influenced these results. Results may have been impacted by administration of MIP interventions and malaria testing outside of study sites, or stock-outs of IPTp occurring unevenly between study arms, although evidence to this effect was not observed. Formal data collection was not undertaken to characterize quality of public sector health records or to track stock-outs. The provision of SP doses and ITNs to sites may limit the generalizability of these results; however, provision was undertaken for all sites and does not appear to be responsible for any difference in SP uptake between study arms. In both Nigeria and Kenya, pregnant women living with HIV infection are prescribed daily oral cotrimoxazole (sulfamethoxazole/trimethoprim) to prevent opportunistic infection, and are consequently not administered IPTp-SP, which may have impacted IPTp-SP administration among study participants. However, the study did not collect data on participants’ HIV status or treatment with cotrimoxazole. While this analysis was not designed for a formal comparison of outcomes between study countries, it is possible that differences in study population (e.g., age, parity) may have contributed to observed differences in the analysis outcomes.

Despite these limitations, findings in this study are notable in that participants in the intervention arm generally received more IPTp doses compared to control. As gains in IPTp coverage have stalled somewhat in many countries in recent years [4], G-ANC may represent a promising approach to improve IPTp adherence, likely related to women’s improved experience of and adherence to recommended ANC contacts. A measurement of health literacy was not undertaken for this analysis. However, recent data suggest that G-ANC improves women’s health literacy on how to prevent and recognize problems, prepare for delivery, and care for their newborn [19].

Further research on G-ANC in LMICs that reports on provision of care is needed, including coverage of MIP interventions for malaria-endemic areas. Alternative service delivery models, e.g., community-based distribution of IPTp-SP, should also continue to be investigated for safety, effective coverage, feasibility, and acceptability. While these findings support further research on the potential benefits and challenges related to this model of ANC, any efforts to reorganize ANC services either within or outside of a research context should be preceded by substantial planning efforts to select facilities with appropriate staffing, adequate supply of commodities (including quality assured SP and appropriate formulations of iron and folic acid), and sufficient ANC client volume to support group enrollment. Group education to ANC clients (e.g., in facility waiting areas) and G-ANC differ substantially in their objectives, content, and approach, and should not be considered interchangeable. Future studies should investigate the potential impact of the G-ANC model on a range of MIP-related process and clinical outcomes.

## Conclusions

Compared to individual ANC, G-ANC may facilitate greater uptake of IPTp-SP and higher use of ITN in the previous night for infants. These findings suggest the G-ANC service delivery platform may have the potential to increase uptake of some recommended MIP interventions.

## Data Availability

The datasets generated and analysed during the current study are available in the Figshare repository, https://figshare.com/articles/Group_Antental_Care_Comparative_Study_Data_and_CodeBook/9744899 [20]

## Abbreviations

ANC: antenatal care
DOT: directly-observed therapy
G-ANC: group antenatal care
IPTp-SP: intermittent preventive treatment of malaria in pregnancy with sulfadoxine–pyrimethamine
ITN: insecticide-treated net
MIP: malaria in pregnancy
WHO: World Health Organization
